# A snapshot of the PD-1/PD-L1 pathway

**DOI:** 10.7150/jca.57334

**Published:** 2021-03-05

**Authors:** Chinmoy Ghosh, Gary Luong, Yue Sun

**Affiliations:** Philips Institute for Oral Health Research, School of Dentistry and Massey Cancer Center, Virginia Commonwealth University, Richmond, VA 23298, USA.

**Keywords:** immune checkpoint, PD-1, PD-L1, PD-L2, T cell, cancer immunotherapy

## Abstract

Cancer cells can evade the attack from host immune systems via hijacking the regulatory circuits mediated by immune checkpoints. Therefore, reactivating the antitumor immunity by blockade of immune checkpoints is considered as a promising strategy to treat cancer. Programmed death protein 1 (PD-1) and its ligand programmed death-ligand 1 (PD-L1) are critical immune checkpoint proteins that responsible for negative regulation of the stability and the integrity of T-cell immune function. Anti-PD-1/PD-L1 drugs have been developed for immune checkpoint blockade and can induce clinical responses across different types of cancers, which provides a new hope to cure cancer. However, the patients' response rates to current anti-PD-1 or anti-PD-L1 therapies are still low and many initial responders finally develop resistance to these therapies. In this review, we provides a snapshot of the PD-1/PD-L1 molecular structure, mechanisms controlling their expression, signaling modulated by PD-1/PD-L1, current anti-PD-1/PD-L1 therapies, and the future perspectives to overcome the resistance.

## Introduction

Over the past ten years, immune checkpoint blockade has revolutionized the treatment for many malignant cancers, providing a new hope to heal cancer patients. Immune checkpoints refer to a set of immune-regulatory pathways maintaining self-tolerance, preventing autoimmunity, and mitigating collateral tissue damages 1-3. Cancer cells can use immune checkpoint pathways to escape from the anti-tumor immune attack 4, 5. Therefore, immune checkpoint blockade can remove the inhibitory signals to unleash the antitumor immune response 2, 6.

T-cell activation is a key step in the immune response initiation and regulation. When confronted by an antigen, effective activation of a naive T-cell and the subsequent immune response requires co-stimulation with “two-signal” from the antigen-presenting cells (APCs) 7, 8. The first signal confers specificity from antigen recognition, provided by the interaction between antigenic major histocompatibility complex (MHC) and T cell receptor (TCR). The second antigen-independent signal is the “costimulatory signal,” delivered by costimulatory molecules expressed on APCs to T-cell. If T- cells receive only antigen-specific TCR stimulation in the absence of co-stimulation, they will become unresponsive (anergic) to subsequent antigenic challenge 9, 10. In a further study, negative costimulatory (i.e., coinhibitory) signals are also found to exist. Immune checkpoint proteins deliver coinhibitory signals to negatively modulate the T-cell activation, which is critical to maintaining self-tolerance and contributes to poor anti-tumor T-cell efficacy 11-13.

Currently, the most understood pathway for T cell co-stimulation is the B7-1, B7-2/CD28 superfamily 14-16. CD28 is a receptor constitutively expressed at the surface of T-cells. B7-1 (CD80) and B7-2 (CD86) are the ligands of CD28 that expressed at the APCs. The binding of B7-1/B7-2 with CD28 provides the primary costimulatory signal from APCs to stimulate T-cell activation 17. Cytotoxic T lymphocyte antigen-4 (CTLA-4) is a CD28 homologue that mediates a negative regulatory effect on T-cell activation 18. The expression of CTLA-4 at T-cells is induced by T-cell activation 19. CTLA-4 binds to B7-1/B7-2 with a much higher affinity (10-20 fold) than CD28 20. The CTLA-4-B7-1/B7-2 binding mediates coinhibitory signal to prevent T-cell activation 21. Therefore, the immune system functions by maintaining an intricate balance between CD28/costimulation-mediated T cell activation and CTLA-4 immune checkpoint-mediated inhibition.

Programmed cell death 1 (PD-1) and its ligand Programmed death-ligand 1 (PD-L1) are identified as another set of the immune checkpoint that mediates coinhibitory signals to T-cell activation 22, 23. PD-1 and PD-L1, together with CTLA-4, are used as important drug targets to develop immune checkpoint blockade therapies. Since 2011, starting with the FDA approval of ipilimumab (an anti-CTLA4 monoclonal antibody) for immunotherapy, immune checkpoint inhibitors targeting the PD-1/PD-L1 axis were also approved to treat a broader range of cancers. The function of PD-1/PD-L1 and CTLA-4 in anti-tumor immune responses are largely distinct 24. CTLA-4 functions at the early stage of T-cell immune response primarily in lymph nodes, whereas PD-1 functions at the later stage of T-cell immune response primarily in peripheral tissues 24.

In this review, we will focus on PD-1/PD-L1 immune checkpoints. We will provide a snapshot of the PD-1/PD-L1 molecular structure, basic biological function, and the usage of anti-PD-1/PD-L1 therapies to treat cancer.

## Molecular Structure and background of the PD-1/PD-L1 pathway

**Figure 1:**
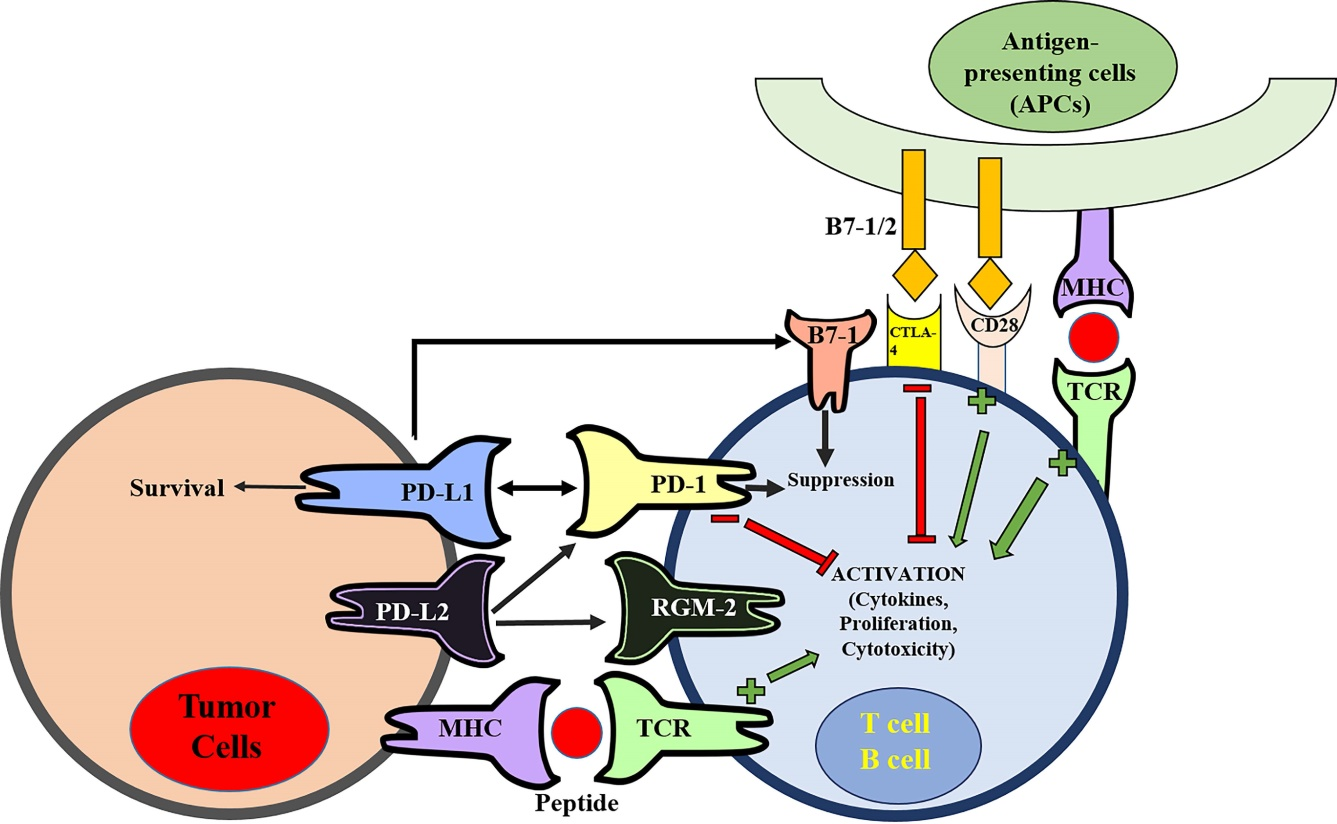
** The pathway of programmed cell death (PD) and Cytotoxic T lymphocyte antigen-4 (CTLA-4) in suppression of T-cell activation.** The MHC-TCR interaction together with B7-1/2-CD28 interaction stimulate T-cell activation. On the contrary, CTLA-4 binds to B7-1/2 and mediates inhibitory signal to prevent T-cell activation. There are at least five interacting molecules in the PD pathway: PD-1, PD-L1, PD-L2, B7-1 (CD80), and RGM-2. PD-L1 and PD-L2 are ligands of PD-1, and the PD-L1/PD-L2 binding with PD-1 leads to suppression of T-cell activation. PD-L1 also interacts with B7-1 (CD80) on activated T-cells to inhibit T-cell activity. PD-L2 has its second receptor RGM-2. The PD-L2-RGM-2 interaction mediates respiratory tolerance.

In 1992, Tasuku Honjo and his colleagues at Kyoto University discovered PD-1, a membrane protein in T-cells that was involved in the cellular process of apoptosis 25. Several other following studies tried to reveal the patterns of PD-1's molecular interaction but it was not until 1999, when a similar B7 homolog, now known today as PD-L1, was observed as an inhibitor of human T-cell responses in-vitro 26. These two critical studies were merged one year later in the year 2000, when Wood Freeman showed that PD-1 is a binding and functional partner of PD-L1 23. The specific function of PD-1 and PD-L1 was unveiled via establishment of gene-knockout mouse strains 2. PD-1-deficient mice developed autoimmune diseases that varied depending on the genetic nature of the mice. Nishimura et al. reported that PD-1 deficiency in C57BL/6 mice led to the occurrence of lupus-like arthritis and glomerulonephritis with IgG3 and C3 deposits 3, whereas the deficiency in BALB/c mice caused fetal dilated cardiomyopathy with collateral production of autoantibodies 4, 5. PD-L1 deficient mice were prone to autoimmune diseases 27. Consistently, the interaction of PD-1 and PD-L1 played a dominant role in the suppression of T-cell responses *in vivo* 26, 28, 29. With this insight, by 2001, another B7 homolog (PD-L2) similar in function to PD-L1 was found to also deliver a suppressive immune response by binding to PD-1 30. Including PD-1, PD-L2 has the ability to interact with other membrane receptors to mediate distinct biological functions 31. PD-L2 can bind with repulsive guidance molecule family member 2 (RGM-2), a molecule that is enriched in lung macrophages. The PD-L2-RGM-2 interaction is required for maintaining the respiratory tolerance 32. Mutagenic study and molecular modeling of PD-L1 and PD-L2 behavior revealed their interaction with B7-1 (CD-80) membrane proteins on activated T-cells, producing the inhibitory immune signal 33, 34. This finding came as a surprise, because B7-1 was previously believed to only act as a functional ligand for CD28 and CTLA-4 35, 36. Therefore, at least five membrane proteins (PD-1, PD-L1, PD-L2, RGM-2, and B7-1) are involved in the PD-1/PD-L1-related pathway (**Figure 1**). Further studies will be required to understand the relative contributions of these molecules during activation or suppression of T cells.

PD-1 is a 288 amino acids protein consisting of an N-terminal IgV domain, a transmembrane domain, a cytoplasmic tail with two tyrosine-based signaling motifs and a 20-amino acids sidechain separating the IgV domain from the plasma domain. PD-1 recruits SH2/SHP3 domain containing proteins with an amino acid sequence (VDYGEL) in the N-terminal domain and a sequence (TEYATI) in the C-terminal domain 37-39. Both sequences form a tyrosine-based immunoreceptor switch motif (ITSM) that is essential for the inhibitory function of PD-1 40. The ligands of PD-1 (PD-L1 and PD-L2) are both type 1 transmembrane glycoproteins containing IgC and IgV domains. They share a 40% amino acid identity, while PD-L1/PD-L2 share a 20% similarity with other B7 homologs. A comparative study between human and mouse PD-L1/PD-L2 orthologs displayed a 70% similarity 39, 41.

PD-1 is present on the surface of B-cells, T-cells and natural killer (NK) cells 42, 43, primarily regulating effector T-cell activity within tissues and limiting their lytic activity in tumors 44. Similarly as found in T- and B-cells, PD-1 is upregulated in dendritic cells (DCs) by various inflammatory stimuli 45. Deficits of PD-1 on DCs enhance anti-bacterial capability 44. Although the PD-1 blockade is more commonly known as an enhancer of effector T cells in tissues and the tumor microenvironment, it may also increase NK cell activity and antibody production through direct and indirect activation of PD-1^+^ B-cells 46.

Previous studies have shown that tumor-associated PD-L1 facilitates apoptosis of activated T-cells 47 and stimulates IL-10 production in human peripheral blood T-cells to promote immune suppression 26. The effects of PD-L1 on immune suppression are known to be much more complex. In addition, to induce apoptosis of T-cells and induction of IL-10, PD-L1 may also induce dysfunction of T-cells through a variety of mechanisms 45. The expression of PD-L1 in tumor cells facilitates apoptosis of activated T-cells via causing T-cells dysfunction and anergy 26, 47-51. In a mouse model of chronic lymphocytic infection, repetitive antigen exposure induced T-cell exhaustion with decreased effector T-cell (Teff) function. Anti-PD-L1 mAb administration reversed this exhaustion and restored Teff function 52. This further supports the function of PD-L1 in down-regulating T-cell activation and in modulating Teff cells. In mouse tumor models, PD-L1^+^ tumor cells are considerably more resistant to CD8^+^ cytolytic T cell (CTL)-mediated destruction than their PD-L1-negative parental cells 53, 89. Ablation of the PD-L1-PD-1 interaction by neutralizing antibodies could restore CTL-mediated killing of tumor cells, suggesting that PD-L1-PD-1 interaction forms a barrier between tumor cells and CTL 53. These PD-L1 functions result in a “molecular shield” on cancer cells that prevents effector immune cells from killing cancer cells 53. Including binding with PD-1, PD-L1 can also interact with B7-1 on the T-cell membrane. B7-1 not only functions as a ligand of CD28 to stimulate T-cell costimulatory signals, but it could also behave as a receptor of PD-L1 to deliver T-cell inhibitory signals 33, 34. The relevance of this PD-L1-B7-1 interaction in tumor immune resistance, however, has not yet been determined.

## PD-1/PD-L1-related cell signaling and the control of PD-1/PD-L1 expression

As the key regulator of immune tolerance and immune exhaustion, the expression of PD-1 is tightly controlled 54. On naïve T-cells, PD-1 is only expressed in a low basal level 55. Initial immune stimulation can induce PD-1 expression on T-cells, B-cells, macrophages, and DCs 54. The gene *Pdcd1* encodes PD-1. A number of transcription factors including NFATc1, FoxO1, AP-1, Notch, STAT3, STAT4, ISGF3, and NF-κB activate the transcription of PD-1 54. On the contrary, T-bet and Blimp-1 are two inhibitory factors that block the PD-1 transcription 54. On CD8^+^ T-cells, PD-1 transcription can be stimulated by TCR signaling 52. Stimulation of TCR activates NFATc1 and AP-1 56-58. These two transcription activators then bind to the cis-regulatory elements of the *Pdcd1* gene to activate PD-1 transcription 54.

As the ligands of PD-1, PD-L1 and PD-L2 are encoded by the *CD274* and *PDCD1LG2* genes separately 59. PD-L1 expression was found in tumor cells, epithelial cells, immune cells, and endothelial cells 8, while PD-L2 expression was primarily observed in antigen-presenting cells (APCs). The PD-L1 expression in cancer can be up-regulated by genetic aberration, transcription control, and post-transcriptional modulation, which contribute to cancer evasion from immune attack 60. PD-L1 genetic copy number gains and amplifications have been found in some types of cancers such as triple-negative breast cancer (TNBC), classical Hodgkin lymphoma (cHL), primary mediastinal B-cell lymphoma (PMBCL), and squamous cell carcinomas of the vulva and cervix 60.

Unlike other members of the CD28 protein family, PD-1 creates a signal only when cross-linked together with a B or T-cell antigen receptor 23, 30. Considered to be a negative regulator of the immune response, PD-1-mediated signaling inhibits T-cell glucose consumption, cytokine production, and cell proliferation by preventing the expression of transcription factors such as GATA-3, T-bet, and Eomesodermin 30, 51, 61. PD-1 ligation also diminishes the phosphorylation of CD3, ZAP70, and protein kinase Cθ 62. In B-cells, PD-1 ligation inhibits Ca^2+^ mobilization and phosphorylation of Igβ, Syk, PLC-γ2, and Erk1/2. These effects are dependent on SHP-2 recruitment by the ITSM motif of PD-1 51. It is worthy to note that CD28 stimulation or IL-2 signaling can override PD-1-mediated inhibition. The effects of PD-1-inhibited ERK activation was found to be nullified through IL-2, IL-7, or IL-15 activation 63.

The recruitment of SHP-1 and SHP-2 proteins via the cytoplasmic tail of PD-1 has been thoroughly documented in human T-cells and B-cells 51, 64. TCR stimulation results in the phosphorylation of tyrosine residues within the ITIM and ITSM motifs in the PD-1 cytoplasmic tail, which recruits SHP-1 and SHP-2 and causes the subsequent dephosphorylation of signaling molecules within proximity downstream of CD28 and the TCR. Therefore, SHP-1 and SHP-2 must play a role in the suppression of T-cell activation. Studies regarding positional mutagenesis have suggested that the ITSM motif is critical for the inhibitory function of PD-1 65. Specifically, the ITSM tyrosine (Y248) of PD-1 binding with SHP-2 is mandatory for PD-1-mediated inhibition of PI3K/Akt activation 65. SHP-2 can interact with phosphorylated ITSM-Y248 residues on two PD-1 molecules to induce PD-1 dimerization, which also promotes the SHP-2 activation 66. SHP-1 is expressed primarily in hematopoietic cells 67. A gene knockout study conducted on SHP-1 deficient mice showed signs of prolonged phosphorylation of the TCR/CD3 complex leading to increased activation of Lck, Fyn, and other proximal TCR signaling proteins 51, 68, 69. Live-cell imaging studies indicate that SHP-2 dephosphorylates PD-1 upon TCR-mediated activation. The same study also found that PD-1 is part of a dynamic T-cell receptor micro-cluster that accumulates at a central supramolecular activation cluster (c-SMAC) 51. Another study conducted on site-directed mutagenesis constructs in Jurkat T-cells expressing mutagenized PD-1, established that only mutated PD-1 Y248 avoided interaction with SHP-2, and that both Y248 and Y223 are actively involved in the inhibitory effects of PD-1 on IL-2 production 47. Although many *in vitro* studies indicate the important role of SHP-2 in PD-1-mediated T-cell suppression, recent *in vivo* study using T cell-specific SHP-2-deficient mice suggests that SHP-2 is dispensable for T-cell exhaustion and for PD-1 signaling 70. Consistently, another recent research indicates that PD-1 can suppress T-cell signaling by a mechanism independent of both SHP-1 and SHP-2 71. These reports suggest that redundant mechanisms other than SHP-1 and SHP-2 may exist to mediate the immune inhibitory function downstream of PD-1.

PD-1 ligation also controls the T-cell cycle. p27kip1, a member of the Kip/Cip family of Cdk inhibitors, abundantly presents in T-cells and interacts with Cdk2 proteins. Ubiquitin-dependent degradation of p27kip1 is required to initiate the cell cycle and the subsequent entry into the S phase by allowing the activation of Cdk2. The Skp1-Cullin-F-box (SCF) family of ubiquitin ligases, SCFskp2, primarily mediates this event 56. TCR/CD3 and CD28 co-stimulation regulates the transcriptional activation of Skp2, the substrate recognition subunit of SCFskp2 ubiquitin ligase, and this process requires simultaneous activation of PI3K/Akt and Ras/MEK/Erk pathways 57. Ligation of PD-1 during T-cell stimulation causes abrogated expression of Skp2, resulting in elevated p27kip1 levels and inhibition of Cdk2 58, 59. The deactivation of Cdk2 disables Rb phosphorylation, consequently affecting its interaction with chromatin remodeling proteins. Cdk2 deactivation also prevents the phosphorylation of checkpoint inhibitor Smad3, upregulating its transcriptional activity 59-61 and increasing the presence of G1 phase Cdk inhibitor, p15INK4B, as well as the loss of Cdk-activating phosphatase Cdc25A 58, 62, 63. Therefore, PD-1 ligation prevents T-cells entry into the S phase.

**Figure 2:**
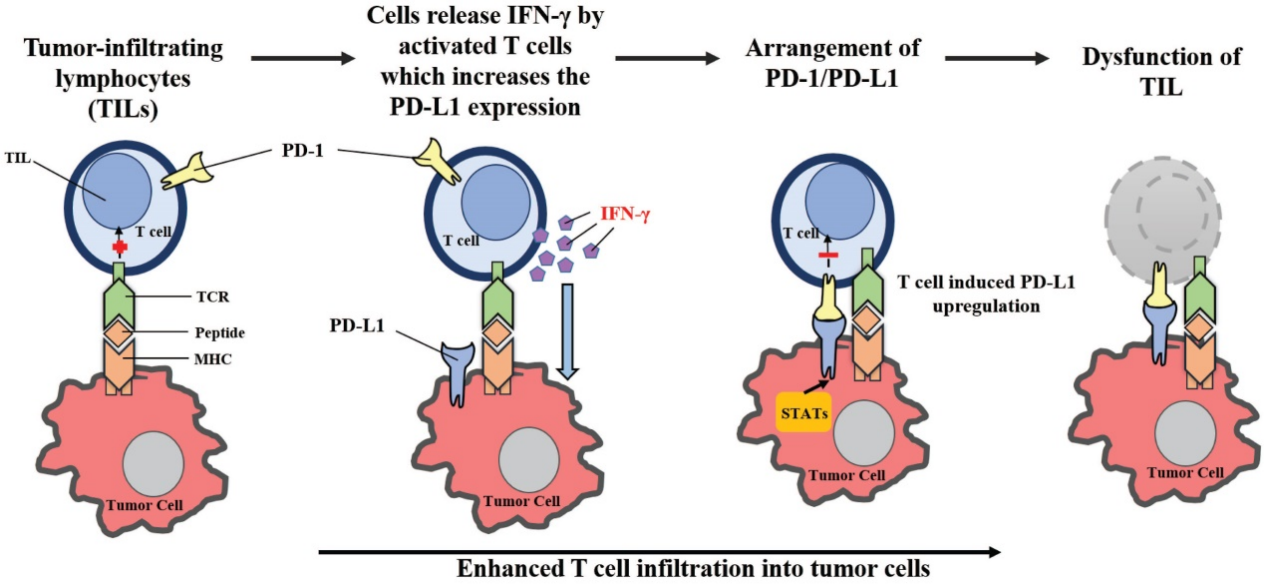
** Adaptive resistance to tumor immunity mediated by PD-1/PD-L1.** Following activation in lymphoid organs, tumor-specific Teffs enter the tumor site to develop TILs. Upon recognition of tumor antigens, TILs release cytokines such as IFN-γ, which stimulates the expression of PD-L1 in the tumor microenvironment. By binding to PD-1, PD-L1 provides a suppressive signal to T-cells and an anti-apoptotic signal to tumor cells, leading to T-cell dysfunction and tumor survival.

By upregulating PD-L1 expression following cancer-induced immune response, the PD-1/PD-L1-mediated evasion of tumor immunity can be described as an “adaptive resistance” (**Figure 2**). While PD-L1 is absent in most normal tissues, its expression can be stimulated by the presence of IFN-γ in virtually any nucleated cell 28, 29, 72, 73. IFN-γ is produced primarily by inflammatory cells of hematopoietic origin, especially by T cells. As a result, in the case of cancer-induced immune response, PD-L1 levels in cancer cells can be upregulated from the exposure to IFN-γ. Tumor-infiltrating lymphocytes (TILs) may develop adaptive resistance through recognition of tumor-specific antigens. These antigens may also be exploited by tumor stromal cells and infiltrating hematopoietic cells, including DCs, neutrophils, macrophages and lymphocytes 2. Upon specific binding of a T cell receptor, TILs release IFN-γ which upregulates PD-L1 expression within the surrounding cells 74. While IFN-γ may enhance TIL effector function through differentiation, the stimulation of antigen presentation in IFN-γ can also downregulate the activation of T-cells by inducing PD-L1 expression 75, 76. On the cell surface, PD-L1 may bind to PD-1 and B7-1, paralyzing T-cells 45. The primary function of PD-L1 upregulation is to prevent instances of inflammation from occurring, and to limit tissue damage. PD-L1 expression within the tumor microenvironment acts as a negative feedback loop to inhibit tumor immunity. Research utilizing laser-captured microdissection and qPCR detected IFN-γ in the presence of TILs and PD-L1+ cells, but was not found within PD-L1- tumors 2. In a mouse tumor model, mAb-mediated neutralization of IFN-γ eliminated PD-L1 upregulation in the tumor microenvironment, proving that IFN-γ is a major inducer of PD-L1 *in vivo* 77. Therefore, the “adaptive resistance” mechanism model helps explain how cancer escapes the immune response, despite endogenous antitumor immune responses. Furthermore, this model helps explain why various cancer immunotherapy approaches fail to control tumor growth or cannot reach the maximal effect.

Although IFN-γ is one of the primary drivers of PD-L1 upregulation, it has been observed in a small fraction of human cases that tumors lacking TILs also express large amounts of PD-L1 without the presence of IFN-γ 78, 79. Upregulation of PD-L1 in cancer cells can be attributed to oncogenic signaling pathways that involve the deletion or silencing of phosphatase tensin homologs (PTEN), constitutive anaplastic lymphoma kinase (ALK), and EGFR 2, 80. Although rare in occurrence, intrinsic oncogenic induction of PD-L1 is only present in roughly 1% of patients with melanoma, but may vary up to 12% in patients diagnosed with lung cancer 78, 79.

The expression of PD-1 or PD-L1 can also be regulated by noncoding RNAs such as microRNAs (miRNAs) and long noncoding RNAs (lncRNAs) 81. miRNAs are a class of small single-stranded non-coding RNAs averagely containing about 22 nucleotides that plays important roles in modulating gene expression. By binding with the 3' untranslational region (3' UTR) of target mRNAs, miRNAs can induce the mRNAs degradation and translational repression. In this way, miRNAs down-regulate their target genes expression. Some miRNAs have been found to target to PD-1 or PD-L1 and regulate their expression levels in cancer cells. For example, miR-28, miR-4717, miR-155, miR-33a, miR-138, and miR-374b modulate PD-1 expression 81. miR-570, miR-513, miR-34, miR-155, miR-140-3p, miR-152, miR-25-93-106b, miR-200, and miR-34 regulate PD-L1 expression 81. In cancers, structural variations such as single nucleotide polymorphisms (SNPs) at the miRNAs binding sites of PD-L1 3' UTR can disrupt miRNAs interaction with PD-L1 mRNA, which leads to elevated PD-L1 expression 82. miRNAs can also indirectly modulate PD-1/PD-L1 expression by modulating their upstream or downstream pathways. miR-197 and miR-3127-5p target to STAT3, a regulator of PD-L1 expression, to indirectly modulate PD-L1 levels 83, 84. miR-21 downregulates JAK2 and STAT1 to inhibit IFN-γ-induced STAT1 signaling 85. In this way, miR-21 decreases PD-1 expression in macrophage. By targeting to phosphatase inhibition and tensin homolog (PTEN), MiR-20b, miR-21, and miR-130b enhance the expression of PD-L1 86.

lncRNAs are a diverse class of transcribed RNAs with a length of more than 200 nucleotides that are not translated into proteins. lncRNAs regulate gene expression via diverse mechanisms 87. The lncRNA small nucleolar RNA host gene 20 (SNHG20) promotes PD-L1 expression in esophageal squamous cell carcinoma (ESCC) 88. Actin filament-associated protein one antisense RNA 1(AFAP1-AS1), another lncRNA, was found to be co-expressed with PD-1 in nasopharyngeal carcinoma (NPC) 89. High expression of AFAP1-AS1 and PD-1 was strongly correlated with distant metastasis and poor prognosis that reveals a novel marker and candidate target for clinical trials 89.

In cancers, other types of noncoding RNAs were also found to be involved in modulating PD-1/PD-L1. A study in colorectal cancer (CRC) highlights the role of circular RNA in accommodating PD-L1 expression, revealing that circular RNA has_circ_0020397 promotes the expression of PD-L1 by inhibiting miR-138 activity 90.

As of 2009, a statistical analysis of renal cancer predicted a worse prognosis relative to PD-L1- tumors 91. Following the study, analysis of various tumors indicated that PD-L1 expression levels might help predict patient survival 73, 92-96. However, considering the broad range of reported outcomes, it is worthy to note that factors ranging from cancer type, stage assessed, IHC technique and treatment history may contribute to variable results associated with the patients' prognosis. Although most studies on PD-1 ligand expression have been focused primarily on PD-L1, PD-L2 is also reported to be positively expressed in various tumors. PD-L2 is upregulated in certain B cell lymphomas, such as primary mediastinal B-cell lymphoma, follicular cell B-cell lymphoma and characteristic in Hodgkin's disease 97. These upregulations are commonly associated with gene amplification or rearrangement with the class II major histocompatibility complex (MHC) transactivator (*CIITA*) locus, a transcriptionally active region in B-cell lymphomas 97.

## Anti-PD-1/PD-L1 therapies in the treatment of cancer

The important role of PD-1/PD-L1 in tumor evasion was first validated in animal models. The treatment with anti-PD-L1 monoclonal antibodies can inhibit the growth of J558L cells, a PD-L1-expressing BALB/c mouse myeloma, in the *in vivo* mouse models 98. In PD-1 deficient mice, the metastasis of B16 melanoma from the spleen to the liver was significantly inhibited 99. These results support that the blockade of PD-1 or PD-L1 has potential to be an effective strategy to treat cancer.

In recent years, more and more therapeutic agents that target PD-1/PD-L1 pathways are developed 81. In **Table 1**, the anti-PD-1 or PD-L1 drugs currently approved by FDA or under clinical trials are summarized. In recent years, anti-PD-1/PD-L1 immunotherapy has shown positive results in clinical applications by inducing regression of tumor growth and metastasis 45. Clinical benefits include possessing durable effects, tolerable toxicity, application to a broad spectrum of cancer types, and effectiveness in solid tumors 100.

In clinical trials involving anti-PD-1, tumor regression was observed in patients with melanoma, renal cell carcinoma, non-small cell lung cancer, and bladder cancer 101, 102. A large phase I clinical trial with the anti-PD-1 antibody MK-3475 was recently shown to lead to response rates of around 38% in patients who had advanced melanoma 103. Another subsequent study reported an overall response rate of 26% in patients who continue to suffer from melanoma after being treated with ipilimumab (a monoclonal antibody of CTLA-4) 6, 104. The phase III trial of nivolumab, a different anti-PD-1 antibody also showed benefits in patients who had metastatic melanoma without a BRAF mutation 105. In this trial, compared with patients treated with dacrabazine chemotherapy, patients treated with nivolumab showed a better objective response rate (40% vs 13.9%) and a better one year overall survival rate (72.9% vs 42.1%) 105. This showed an overall improvement in survival for patients treated with nivolumab as compared to patients treated with docetaxel chemotherapy. In December 2014, the FDA approved nivolumab as a treatment for patients with metastatic melanoma and in the following year, approved nivolumab clinical application for patients with previously treated advanced or metastatic non-small cell lung cancer 6.

A number of companies developing and testing antibodies targeting PD-1 or PD-L1 currently have no valid information on their clinical performance. Theoretically it is possible that a PD-1 antibody would prevent PD-1 from interacting with both PD-L1 and PD-L2 but not the interaction between PD-L1 and B7-1 2. Most PD-L1 antibodies block the interaction between PD-L1 and B7-1 and between PD-L1 and PD-1 but would not block PD-1 from interacting with PD-L2. Therefore, it is possible that PD-1 and PD-L1 antibodies will not have redundant activity depending on which interactions dominate in a particular cancer.

The response rates to current anti-PD-1 or anti-PD-L1 therapies are still very limited. Therefore, it is urgently required to identify biomarkers that can indicate the patients' response. Initial phase I trials with anti-PD-1 therapy (nivolumab) reported that PD-L1 expression in tumor cells, measured on pretreatment archival samples by immunohistochemical (IHC) methods, could potentially serve as a predictive biomarker, broadening the range of treatable patients 95. Patients with PD-L1 positive tumors (≥ 5% staining for PD-L1 on tumor cells) had an objective response rate of 36% (9 of 25 patients) whereas patients with PD-L1 negative tumors did not show any objective clinical responses (0 of 17 patients) 6. Although PD-L1 expression in tumor tissues has been correlated to higher response rates, its clinical benefits have yet to be proven. Recent studies suggest that the PD-L1 expression status of tumor-infiltrating immune cells (TIICs) may also serve as a biomarker in predicting immunotherapy response in different patient subgroups. In a phase I study of anti-PD-L1 mAb (Atezolizumab), urothelial bladder cancer patients with tumors containing PD-L1-positive TIICs shows a higher response rate to this immunotherapy 106.

## Combination therapy for clinical benefits against cancer

Combination therapy has been an option to improve the response rate of cancer patients to anti-PD-1/PD-L1 treatment. The main principle for most combination therapies is to boost the efficiency of PD-1/PD-L1 blockade by either improving tumor antigen presentation or rescuing dysfunctional immune effector cells. Combining anti-PD-1/PD-L1 therapy with other immune checkpoint inhibitors, cancer vaccines and immune-stimulatory agents, chemotherapy, and radiotherapy has shown some success in various types of cancers 107, 108.

Preclinical studies in murine models indicate that CTLA-4 and PD-1 regulate distinct inhibitory pathways consisting of non-overlapping mechanisms of action, suggesting combination therapy with both might be more effective than either one alone 109, 110. Early studies that used conventional KO mice demonstrated that the major role of CTLA-4 lies in regulating the T-cell response to self-antigens because CTLA-4-KO mice spontaneously produce massive infiltrating T-cells to normal organs. These tissue-infiltrating T-cells are highly active and eventually result in death by causing damage to normal tissue without specific antigen exposure 11, 111. Consistently, anti-CTLA-4 mAb can induce various types of autoreactive T-cell responses in various mouse models 112, 113. Mice with Treg selective ablation of CTLA-4 reiterate the majority of autoimmune phenotypes found in conventional KO mice, including tissue-infiltrating T-cells, harming normal tissues and organs 114. Therefore, the physiological function of CTLA-4 appears to be suppressing T-cell responses to self-antigens while controlling Treg activity.

In contrast to CTLA-4 KO mice, PD-L1 KO mice do not spontaneously develop inflammation within their organs, and experience no substantial changes to life span 115. However, PD-1 KO mice typically develop strain-specific autoimmune diseases in the span of a few months 116. Both PD-1 and PD-L1 KO mice are prone to autoimmune diseases because severe symptoms develop when they are immunized with autoantigens 115. As PD-L1 has a limited distribution in normal tissues and is largely an inducible molecule controlled by IFN-γ, its major physiological functions lie in mediating negative feedback in tissue inflammation. In the context of tumor growth, which is often promoted by chronic inflammation, anti-PD-1/PD-L1 therapies selectively modulates inflammatory T-cell responses at the tumor site and inhibits PD-L1 induced tumor function. Thus, the primary physiological function of the PD-1/PD-L1 pathway is to control ongoing inflammatory responses and prevent the spread of inflammation, rather than the systematic regulation of autoreactive T-cell responses. These attributes place anti-PD therapy in a different category scientifically and practically from anti-CTLA-4 therapy.

Substantial data suggests the possible benefits by combining anti-CTLA-4 and anti-PD-1/PD-L1 therapies. Anti-CTLA-4 has been found to accelerate T-cells infiltration into tumors, which resulted with an increase in the number of T-cells and a concomitant increase in IFN-γ production 117. This can induce expression of PD-L1 in the tumor microenvironment, with subsequent inhibition of anti-tumor T-cell responses. However, it can enhance the chance of benefit from anti-PD-1 and anti-PD-L1 therapies. Thus, the combinative treatment with anti-CTLA-4 plus anti-PD-1 or anti-PD-L1 should lead to the creation of an immunogenic tumor microenvironment with clinical benefits for patients regardless of their tumor original PD-L1 expression levels. From a recent phase I clinical trial, data indicated the administration of anti-CTLA-4 (ipilimumab) and anti-PD-1 (nivolumab), in patients with metastatic melanoma resulted in similar response rates in the setting of concurrent therapy regardless of PD-L1 expression in pretreatment tumor tissues 118. For patients with PD-L1-positive tumors, the objective response rate was 46% (6 of 13 patients), similar to a response rate of 41% (9 of 22 patients) in patients with PD-L1-negative tumors. For a combinatorial study with anti-PD-1 (nivolumab) and anti-CTLA-4 (ipilimumab) in patients with metastatic renal cell carcinoma (mRCC), similar data was reported 6.

Conventional cancer therapies such as radiotherapy and chemotherapy can induce tumor cell death to promote tumor antigens release. Furthermore, they can activate T-cells and enhance T-cells migrate into tumor tissues 119. Thus, combination of conventional therapies with immune checkpoint therapies should create an “immunogenic” tumor microenvironment effective in patients. A number of ongoing trials combining radiation therapy and anti-PD-1/PD-L1 mAbs provide valuable information regarding schedule, safety, and efficacy in these combinations for future studies 120.

In radiotherapy, cancer cells are generally killed by radiation. However, accumulating evidence suggests it also has systemic effects such as reverting tumor suppressive barriers in the tumor microenvironment, and indirectly stimulating the immune system by releasing tumor antigens 121, 122. In clinical trials and preclinical models, radiotherapy has shown synergy with various immunotherapeutic treatments, providing primary rationale to combine radiotherapy with other immunotherapies 123. Combining radiotherapy with PD-1/PD-L1 blockade therapy in pre-clinical studies activated cytotoxic T lymphocytes (CTLs) and reduced myeloid-derived suppressor cells 123. These results propelled two open phase III trials of Nivolumab combination with radiotherapy for NSCLC (NCT02768558) and glioblastoma (NCT02617589), currently still ongoing 121.

Chemotherapy eradicates cancer cells by targeting its DNA synthesis and replication process. Recent reports however suggest that it also has several off‐target effects. One particular effect is the priming of tumor‐specific T-cells by increasing the presence of tumor antigens following cell death, and subverting immunosuppressive factors 124. In consideration of these chemotherapeutic off-target tendencies, appropriate combination of chemotherapy drugs with PD‐1/PD-L1 blockade could augment the efficacy of the anti-PD-1/PD-L1 therapies, particularly in less immunogenic, chemo‐sensitive tumors. A recent phase II clinical study reported that pembrolizumab combined with pemetrexed/carboplatin enhanced the efficacy of chemotherapy alone for the treatment of metastatic non-squamous NSCLC, and has been approved by FDA (KEYNOTE‐021) 121, 125.

Other combinational therapies under development utilize the blockade of multiple inhibitory pathways, such as LAG-3 126, TIM-3 127, VISTA 128, BTLA 129, and oncolytic virus 130. The development of these therapies remain critical in inducing immune antitumor responses in cancer patients, even for individuals who have been diagnosed with non-immunogenic or PD-L1-negative tumors. Although major histocompatibility complex II (MHC-II) has been previously reported to be the ligand of the coinhibitory receptor LAG3, LSECtin has also been reported to be an additional ligand 131, 132. LSECtin is expressed by liver and tumor cells and may account for the biological role of LAG3 in CD8^+^ and natural killer (NK) cells, as neither cell type interacts with MHC-II. Further complexity has been observed in the context of the coinhibitory receptor TIM3, as four more ligands have been reported to date: Galectin-9, PtdSer, HMGB1, and CEACAM1 132, 133. The underlying mechanisms of ligand regulation, however, whether they affect each other's binding, and whether each ligand leads to unique downstream signaling events remains unclear. Furthermore, although TIM3 is thought to be a primary marker of T-cell activation and exhaustion, TIM3 also functions to attenuate NK cell cytotoxicity 134. This suggests that other costimulatory molecules like TIM3 may have significant biological functions in a multitude of other cell types. VISTA, another suppressor of T-cell activation, presents yet another ambiguity. Studies have indicated VISTA is most likely both a ligand with an unknown receptor on APCs (homologous to PD-L1) and a receptor on T-cells with an unknown ligand 134, 135. Furthermore, the biological roles of several B7 ligand family proteins, including their counter-receptors, also remain to be understood. B7-H3 is believed to have both costimulatory and coinhibitory functions, likely dependent on the context of expression 136, 137, but both the receptor and molecular interactions of B7-H3 in posttranscriptional regulation remain unknown.

Oncolytic viruses (OVs) replicate selectively in cancer cells and kill tumor cells without inducing damage to normal tissue 138. Known to be effective for advanced melanoma, OVs promote innate and adaptive antitumor immunity 139. Development of therapy combining T‐VEC, oncolytic virus therapy, and pembrolizumab is currently undergoing a phase I clinical trial 140, 141. Recent data indicates objective tumor response compared to applying pembrolizumab alone (NCT02263508) but further trials are necessary to establish this as a standard option 142.

A key factor in melanoma proliferation is BRAF mutation. The small molecule inhibitors targeting mutant BRAF induces a significant clinical response in specific patients, but remain limited in other cases 143. It is believed that PD‐L1 upregulation is associated with the development of resistance to BRAF inhibitors, providing a rationale for possible combinative therapies. This insight has paved the way for a fruitful approach of combining PD‐1/PD-L1 blockade with BRAF inhibition in preclinical models. Whilst several clinical trials are ongoing (NCT01656642 and NCT02224781), the success rates have not been promising (NCT02027961) 144, 145. Vascular endothelial growth factor (VEGF) is an angiogenic factor that influences growth and survival in the vascular endothelium. Although VEGF inhibitors are used for preventing angiogenesis and/or normalization of vascular permeability in tumor microenvironment, VEGF inhibition also promotes the differentiation and function of immune cells 146. Moreover, certain types of VEGF receptors are also expressed by DCs, macrophages and lymphocytes, evoking immune suppression 147. Initial studies of the relationship between VEGF signaling and the immune system suggest the combination of PD‐1/PD-L1 blockade with VEGF inhibition have the potential to enhance the efficiency of PD‐1/PD-L1 blockade 148.

## Figures and Tables

**Table 1:** Summary of the clinical development of therapeutic agents that target PD pathways in clinical trials

Target	Ligand	Biological function	Therapeutic agent	Class	State of clinical development
PD-1	PD-L1, PD-L2	Negative T-cell costimulation (primarily at priming); attenuate peripheral activity, preserve T-cell function in the context of chronic antigen	Niovolumab [MDX-1106 (also known as BMS-936558)	Human IgG4	Phase I/II trials in patients with melanoma, renal cell carcinoma, Hodgkin's lymphoma, Head and neck cancer, urothelial carcinoma, hepatocellular carcinoma and lung cancers.
			Pembrolizumab (MK3475)	Humanized IgG4	Phase I trial in multiple cancers like melanoma and for metastatic nonsmall-cell lung cancer (NSCLC), gastric cancer.
			CT-011	Humanized IgG1k	Phase I/II trial in multiple cancers
			AMP-514 (MEDI0680)	PD-L2 IgG2a fusion protein	Phase I/II trial in multiple cancers
			PDR001	Humanized IgG4	Phase I/II trial in multiple cancers
			Cemiplimab	Human IgG4	Pre-Registered in Metastatic CSCC; NSCLC; Cervical Cancer
			Tislelizumab	Humanized IgG4	Phase III trial in unresectable HCC
			Sintilimab (IBI-308)	Humanized IgG4	Phase II trial in NSCLC
			Spartalizumab	Humanized IgG4/k	Phase II trial in advanced melanoma
			Atezolizumab	Humanized IgG1k	Phase II trial in NSCLC
			Camrelizumab	Humanized IgG4	Phase II trial in Non-squamous NSCLC and squamous esophageal Cancer NSCLC
			SHR-1210	Humanized IgG4	Phase II trial in Gastric Cancer
			Cetrelimab (JNJ-63723283)	IgG4	Phase I/II trial in Multiple Myeloma; Castration-Resistant Prostatic Neoplasm
			TTI-622	Humanized IgG4	Phase I trial in Lymphoma; Myeloma
			HLX10	Humanized IgG4	Phase I trial in Solid Tumor
			PF-06801591	Humanized IgG4	Phase I trial in Solid Tumor; Prostatic Cancer; Melanoma; Ovarian Cancer; Sarcoma; Hodgkin lymphoma
PD-L1	PD-1, B7-1 (CD80)	Attenuate T-cell activity in inflamed peripheral tissues	Durvalumab (MEDI4736)	Humanized IgG1	It is approved by FDA for treatment of metastatic urothelial carcinoma
			Atezolizumab (MPDL-3280A)	Human IgG1	FDA approved for treatment of metastatic NSCLC
			Avelumab (MSB0010718C)	Fully Humanized IgG1	FDA approved for treatment of metastatic Marked cell carcinoma (MCC)
			MDX-1105/BMS-936559	Human IgG4	Phase I/II trial in multiple cancers
			M-7824	Fully Humanized IgG1	Phase II trial in Advanced solid tumors
			CX-072	Humanized IgG4	Phase II trial in Solid tumors; Lymphomas
			MSB-2311	Humanized IgG4	Phase II trial in Solid tumors
			FS-118	Human IgG1	Phase II trial in Advanced cancer
			LY-3300054	Human IgG1	Phase I trial in Solid tumors
